# Effects of Temperature, Precipitation, and Sunshine on Cold-Tolerant Wheat Yield Under Warming Trends: A 20-Year Study in Hokkaido, Japan

**DOI:** 10.3390/plants13223165

**Published:** 2024-11-11

**Authors:** Zenta Nishio, Masatomo Kurushima, Takeshi Suzuki, Seiji Shimoda, Tomoyoshi Hirota

**Affiliations:** 1 Department of Agriculture, Tokyo University of Agriculture, Atsugi 243-0034, Kanagawa, Japan; 2 Hokkaido Research Organization (HRO), Tokachi Agricultural Experimental Station, Memuro 082-0081, Hokkaido, Japan; 3 National Agricultural Research Organization (NARO), Hokkaido Agricultural Research Center, Memuro 082-0081, Hokkaido, Japan; 4 Graduate School Bioresource and Bioenvironmental Sciences, Kyushu University, Nishi-ku, Fukuoka 819-0382, Fukuoka, Japan

**Keywords:** cold-tolerant wheat, global warming adaptation, snow-cover reduction, wheat phenology changes, grain-filling period extension, high summer temperature mitigation

## Abstract

To clarify the adaptation strategies of cold-tolerant wheat against global warming, this study examined the effects of daily temperature, precipitation, and sunshine duration on wheat yield in Hokkaido, Japan, over 13 years (2011–2023). Yield components were also analyzed over 20 years (2004–2023). The number of snow-cover days decreased by about 24 days over the 20-year period. As a result, the growth of overwintered wheat accelerated, with the heading and maturity of plants advancing by about 8 and 5 days, respectively, and the grain-filling period extending from about 44 to about 48 days. Multiple regression analysis was conducted using wheat yield as the objective variable and weather conditions as explanatory variables. Three weather conditions were selected: precipitation for 8 days from 27 March, sunshine hours for 8 days from 21 March, and sunshine hours for 12 days from 13 June, which yielded a coefficient of determination of 0.953. Despite the highest mean summer temperatures on record being registered in 2023, high yields were ensured by the number of sunshine hours, which were approximately 1.5 times the normally recorded hours. This highlights the importance of this parameter in mitigating the impact of high summer temperatures.

## 1. Introduction

Hokkaido, the northernmost region of Japan, is an important granary area that accounts for about 70% of the domestic wheat production. It is located in the East Asian monsoon climate zone between 42° and 45° N and experiences intense snowfall in winter as a result of the northwest monsoon passing over the Sea of Japan from the Siberian region, while in summer it is strongly influenced by the hot and humid southeast monsoon passing over the Pacific Ocean. Therefore, the major threats to wheat in Hokkaido are snow mold disease, which occurs after more than 4 months of snow cover, and Fusarium head blight and pre-harvest sprouting damage, which are caused by unseasonable weather from the flowering period to around harvest time in the summer [1,2].

**Figure 1:**
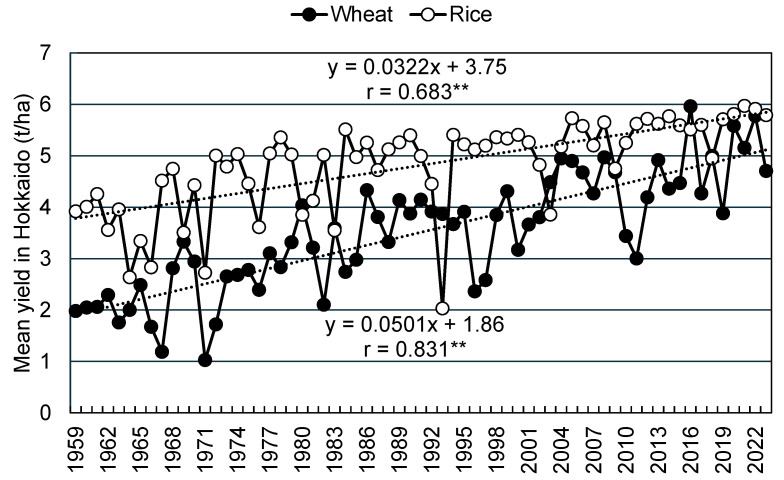
Trends in wheat and rice yields in Hokkaido from 1958 to 2023. The dashed lines represent the liner regression curve. ** *p* < 0.01.

In the past, wheat in Japan was often grown as a winter crop in rice paddies with poor drainage, and wheat yield per area was only about half that of paddy rice until the 1990s. To improve the self-sufficiency rate of domestic wheat, vigorous efforts have been made to enhance wheat varieties and develop cultivation techniques, mainly in Hokkaido. As a result, in this region, wheat yields have continuously increased at a higher rate compared to paddy rice yields and have recently exceeded them in some years (Figure 1) [3]. Alternatively, wheat yields are more variable and unstable than those of paddy rice. In particular, since 2010, high summer temperatures and low sunlight have shortened the growing season and reduced the size of seedlings, and weather stresses, such as rainfall and unseasonable weather conditions after the flowering period, as well as typhoons, have frequently reduced crop yields. These weather phenomena are believed to be the result of global warming.

Sofield et al. [4] has reported that higher temperatures accelerate the rate of wheat maturation and significantly shorten the grain-filling period, affecting yield. Fisher [5] found that temperature and sunlight before wheat heading had a significant effect on grain number, and Tashiro and Wardlow [6] reported that a 1 °C increase in temperature during wheat grain-filling period reduced grain weight by about 5%. Porter and Gawith [7] emphasized the importance of modelling the combined effects of extreme weather, such as the impact of high temperature on grain set. These and several subsequent large-scale studies in wheat-growing regions around the world have shown that wheat yields are likely to be significantly reduced by possible future increases in temperature [8,9]. Shimoda and Sugikawa [10] pointed out that the combination of delayed flowering due to delayed growth and lack of sunlight in the early stage of grain filling results in more severe yield losses in high-yielding wheat varieties than in normal varieties. In addition, Shimoda et al. [11] analyzed the weather conditions contributing to the “yield gap”, which is the gap between potential yield (PY; the maximum yield that can be obtained under ideal environmental and growing conditions) and the actual yield of wheat, in Hokkaido for about 40 years since the mid-1980s. In this region, new wheat varieties have been introduced approximately every decade. Initially, these varieties were developed with improved resistance to pre-harvest sprouting, which frequently occurs under humid conditions at harvest time. Subsequently, they were bred for enhanced resistance to Fusarium head blight, which develops under humid conditions during the grain-filling period. As a result, the period during which the yield gap was affected by the vapor pressure deficit (i.e., a measure of the additional grams of water vapor that can be contained in 1 m^3^ of air) was shown to have shifted from the harvest period to the flowering period.

In 2023, wheat growers were shocked to learn that the entire world, including Hokkaido, experienced record high temperatures. To address this issue, it is urgent to analyze the resulting impacts using the latest crop growth and weather data and to consider new adaptation measures to mitigate the effects of climate change. Recently, Nishio et al. [12] utilized daily meteorological data to elucidate in detail the relationships between wheat yield and climatic conditions over the past 20 years in northern Kyushu, Japan, a warm region of East Asia. In the present study, we employed a similar methodology to analyze the weather response of wheat in Hokkaido, a cold region of East Asia, using the latest data from a 13-year period spanning 2011 to 2023. This analysis aims to contribute to the development of recent climate change adaptation strategies. During this time, the high-yielding wheat variety Kitahonami, which is currently the most cultivated wheat in Hokkaido, was prevalent. This analysis, unparalleled in terms of the level of detail, investigated the effects of weather conditions on wheat growth, yield, and yield components by incorporating comprehensive data on daily temperature, precipitation, and sunshine hours.

**Figure 2:**
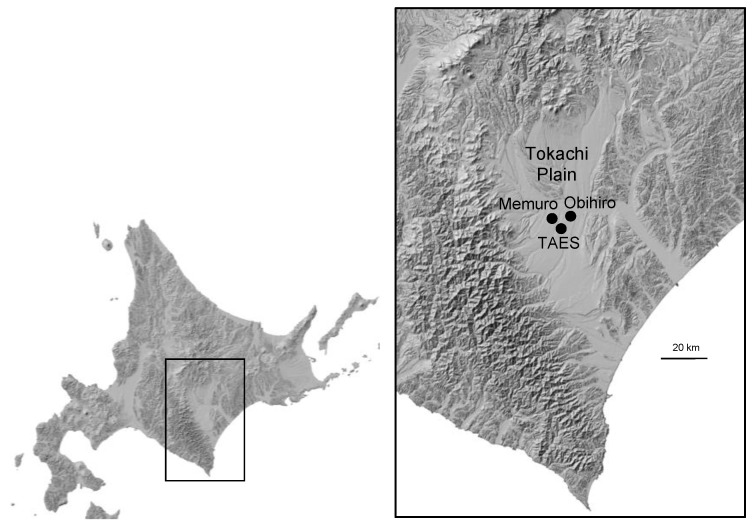
Tokachi Plain and location of Tokachi agricultural experimental station (TAES) and automated meteorological data acquisition system (AMEDAS) in Obihiro and Memuro. The black dots indicate the respective locations.

The region examined in this study is the Tokachi Plain, which is the largest wheat-producing area in Japan and accounts for approximately 40% of Hokkaido’s wheat production (Figure 2). Several previous studies have examined the relationship between wheat and weather in the Tokachi Plain. In this regard, a significant advantage of our study is the ability to identify trends and variations in the responses to weather by comparing our findings with those of previous studies [10,11,12,13,14,15]. The recent rise in temperatures has exacerbated these conditions, making Hokkaido a particularly problematic region for wheat cultivation, as wheat prefers cool and dry weather during the growing season.

## 2. Results

### 2.1. Trends in Weather Conditions and Wheat Growing Seasons

**Figure 3:**
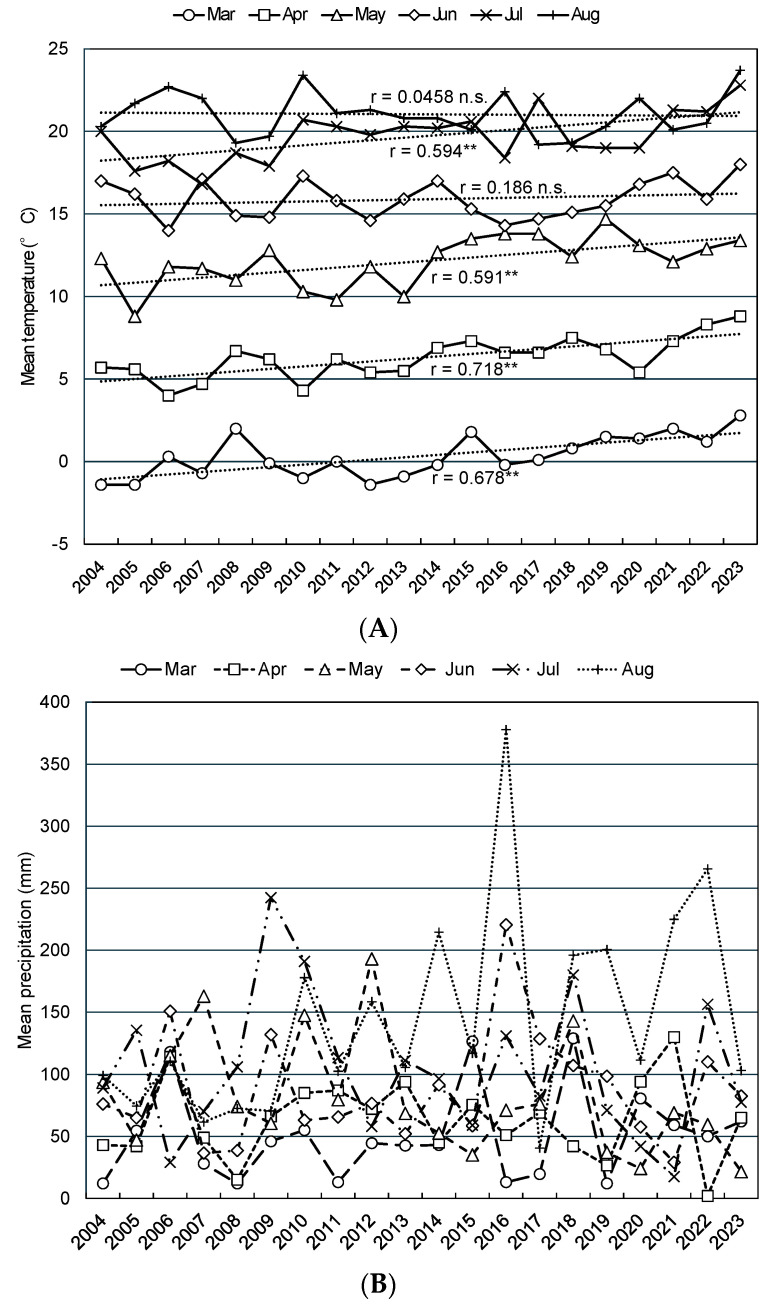

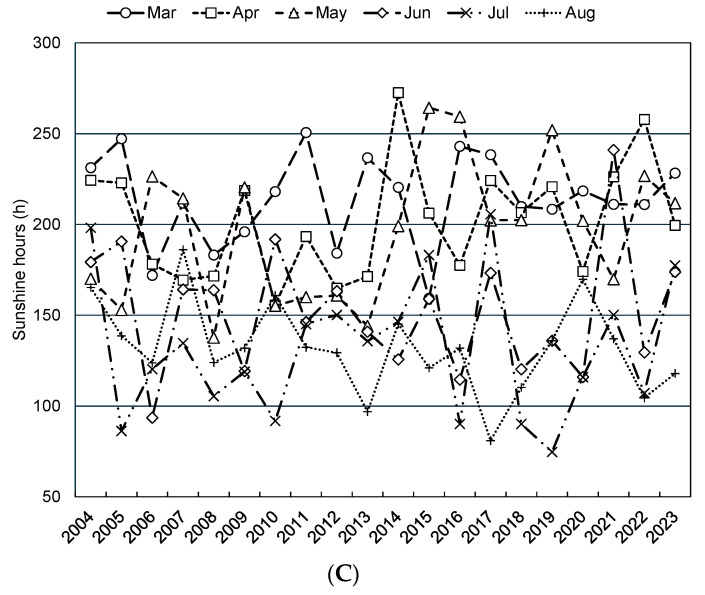
Trends in monthly mean temperature (**A**), precipitation (**B**), and sunshine hours (**C**) in the Tokachi Plain (Obihiro) from 2004 to 2023. The dashed lines represent the liner regression curve. ** *p* < 0.01, n.s. not significant.

Figure 3 depicts the monthly mean temperature, mean precipitation, and mean sunshine duration from March to August, after the snow-cover period, for the past 20 years from 2004 to 2023 for Obihiro, which is located in the center of the Tokachi Plain. Among the monthly mean temperatures, those in March, April, May, and July increased each year and were significantly positively correlated with year, while those in June and August showed no significant correlations with year. Conversely, monthly mean precipitation was outstandingly high (378 mm) in August 2016, when four typhoons hit or approached Hokkaido for the first time in recorded history. The coefficient of correlation between August precipitation and year was 0.462 (*p* < 0.05, *n* = 20), which was the only statistically significant positive correlation among monthly precipitation amounts. In contrast, no significant correlation was found between monthly sunshine hours and year.

**Figure 4:**
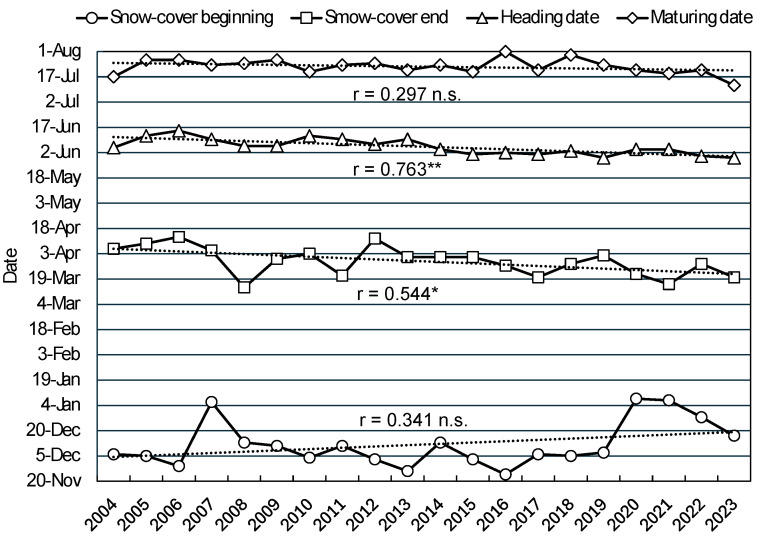
The beginning and end of the snow-cover period in Memuro and the transition of heading and maturity dates of the wheat cultivar Kitahonami from 2004 to 2023. The dashed lines represent the liner regression curve. * *p* < 0.05, ** *p* < 0.01, n.s. not significant.

Figure 4 shows the Kitahonami growth seasons (heading and maturity) and the beginning and ending dates of the snow-cover period for each season recorded at the TAES from 2004 to 2023. The 5-year moving mean of these periods indicated a significant negative correlation between the heading and maturity dates for this wheat cultivar and each year, with these phases advancing by approximately 8 days and 5 days, respectively, over the 20-year period. The grain-filling period, which is defined as the interval between heading and maturity, exhibited a positive, though not statistically significant, correlation with year (r = 0.398, *p* < 0.10), with the 5-year moving mean increasing from 44 to 48 days. The date marking the start of the snow-cover period did not show a significant correlation with year; however, the end date as well as the duration of this period were significantly negatively correlated with it. The 5-year moving mean of the snow-cover end date advanced by approximately 10 days, and the duration of the snow-cover period was shortened by about 24 days over the 20-year period examined. During this time, the last day of the snow-cover period, which marks the start of wheat growth after overwintering, moved forward, resulting in an earlier heading phase. Conversely, the maturity period did not advance as much, resulting in a higher number of days between the heading and maturity phases.

### 2.2. Relationship of Growth Data and Yield Components with Mean Wheat Yield in the Tokachi Plain

The correlation between mean wheat yield in the Tokachi Plain and data from yield trials at the TAES is shown in Table 1. The mean wheat yield in Tokachi for 2011–2023, when this analysis was conducted, showed a strong positive correlation (r = 0.890, *p* < 0.01, *n* = 13) with the yield achieved at the TAES. The two yields also showed significant positive correlations with the number of kernels per spike and spike weight and a significant negative correlation with the maturity stage. The number of spikes per m^2^ was not significantly correlated with yield, but did show significant negative correlations with thousand-kernel weight, number of kernels per spike, and single-spike weight. A negative correlation, although not statistically significant, was also observed between yield and number of spikes per m^2^.

Multiple regression analysis based on the method of decreasing variables (significance level *p* = 0.01) was conducted using mean wheat yield in the Tokachi Plain as the objective variable and the yield components obtained at the TAES as explanatory variables. As a result, spike number and single-spike weight were selected as significant explanatory variables, and the following regression equation was obtained;

Wheat yield in the Tokachi Plain (kg/10a) = 0.4287 × (spike number) + 513.56 × (single spike weight) − 314.54

The adjusted coefficient of determination for this regression equation was 0.770, indicating that these two yield components (spike number and single-spike weight) explained 77% of the variation in wheat yield.

### 2.3. Relationship Between Weather Conditions and Wheat Yield and Its Components

**Figure 5:**
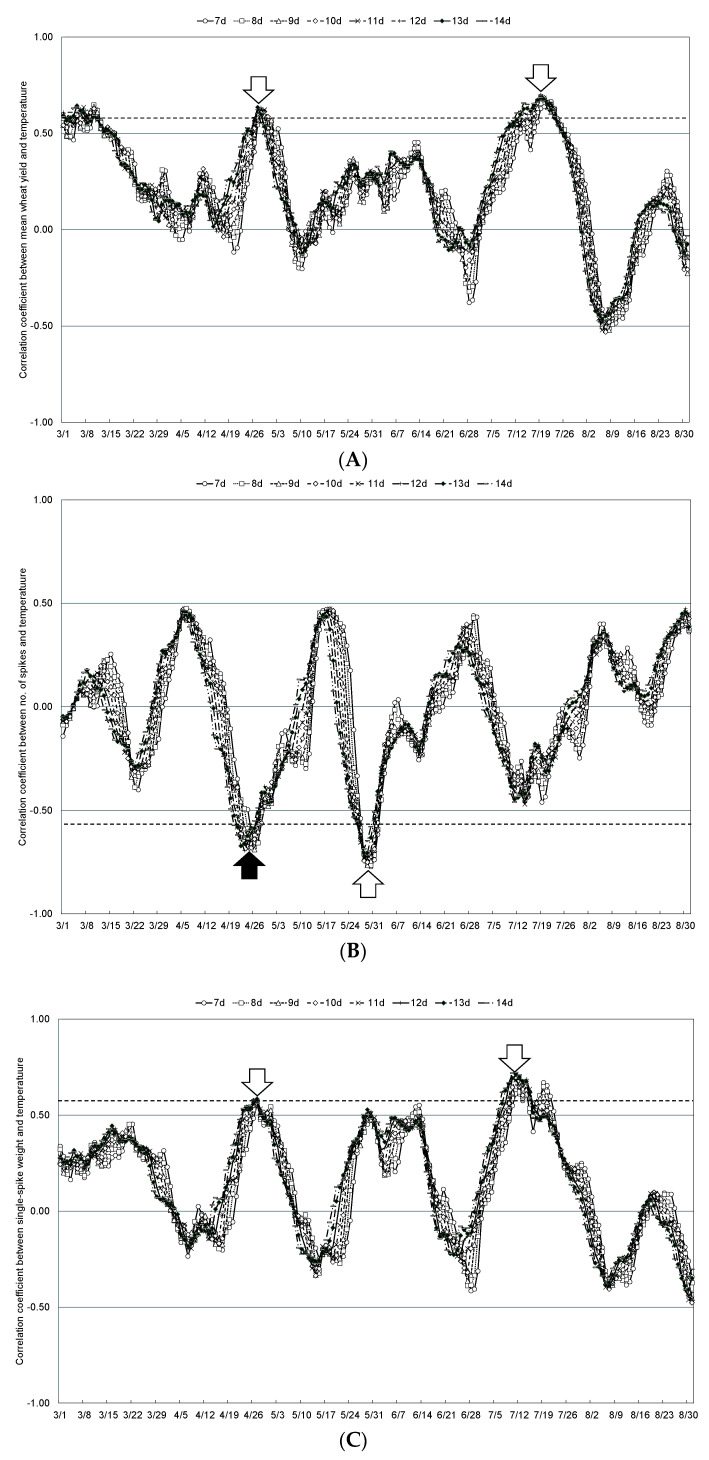

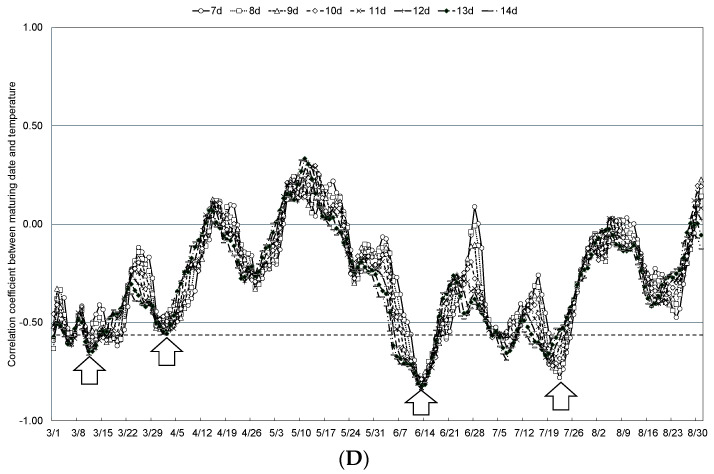
Correlation coefficients between the moving mean temperature over 7 to 14 days and mean wheat yield in the Tokachi Plain (**A**), number of spikes (**B**), single-spike weight (**C**), and maturing date (**D**) from 2011 to 2023. Plots above the dashed line for positive values or below the dashed line for negative values indicate statistical significance at the 1% level. White arrows indicate weather conditions that are statistically significantly correlated, and black arrows indicate weather conditions that were selected as statistically significant explanatory variables.

**Figure 6:**
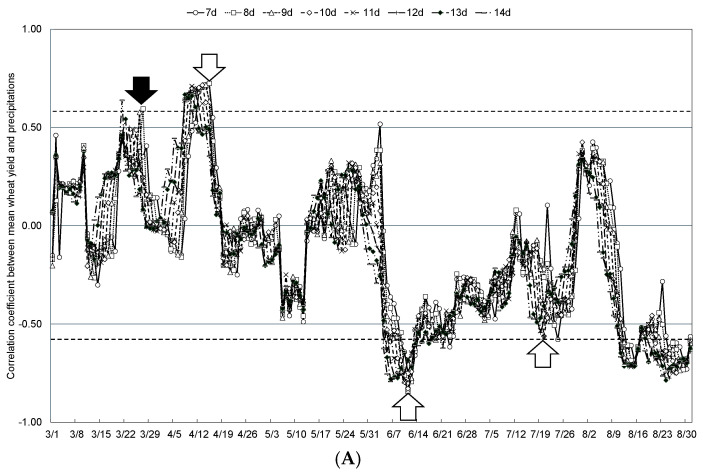

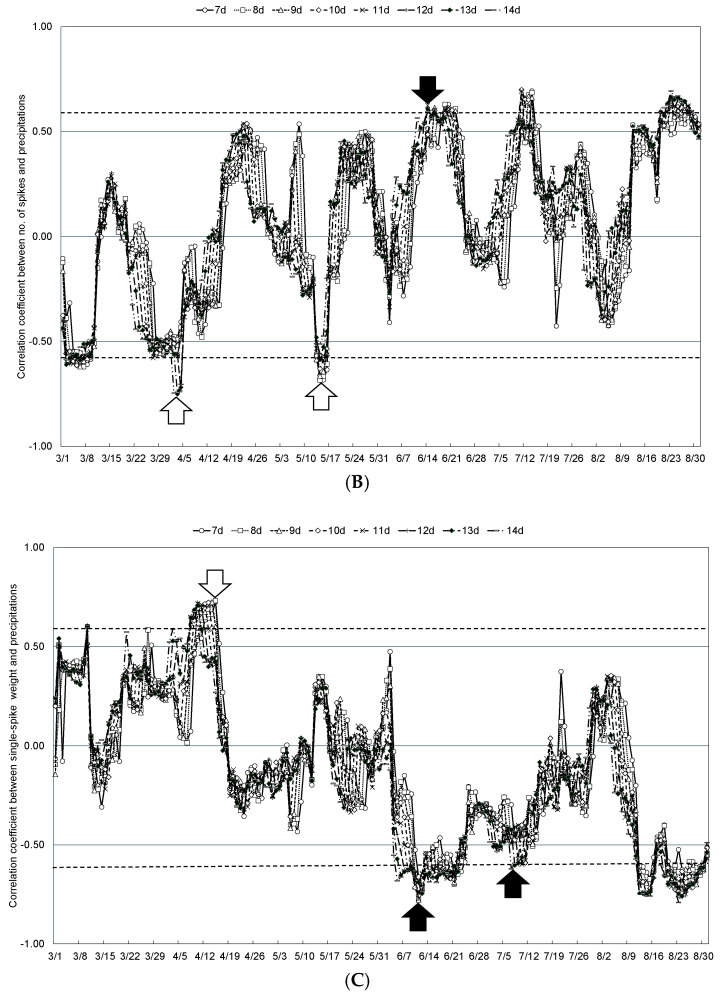

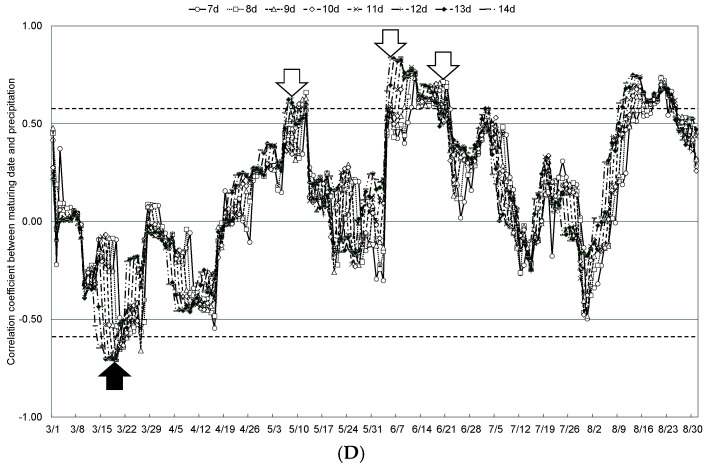
Correlation coefficients between the moving mean precipitation over 7 to 14 days and mean wheat yield in the Tokachi Plain (**A**), number of spikes (**B**), single-spike weight (**C**), and maturing date (**D**) from 2011 to 2023. Plots above the dashed line for positive values or below the dashed line for negative values indicate statistical significance at the 1% level. White arrows indicate weather conditions that are statistically significantly correlated, and black arrows indicate weather conditions that were selected as statistically significant explanatory variables.

**Figure 7:**
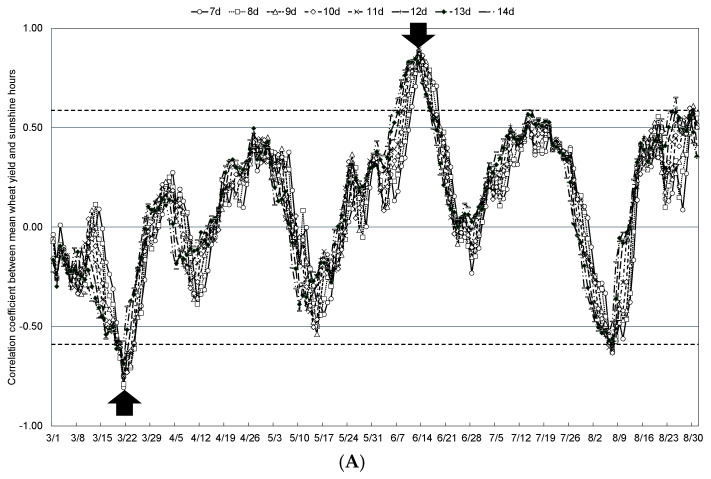

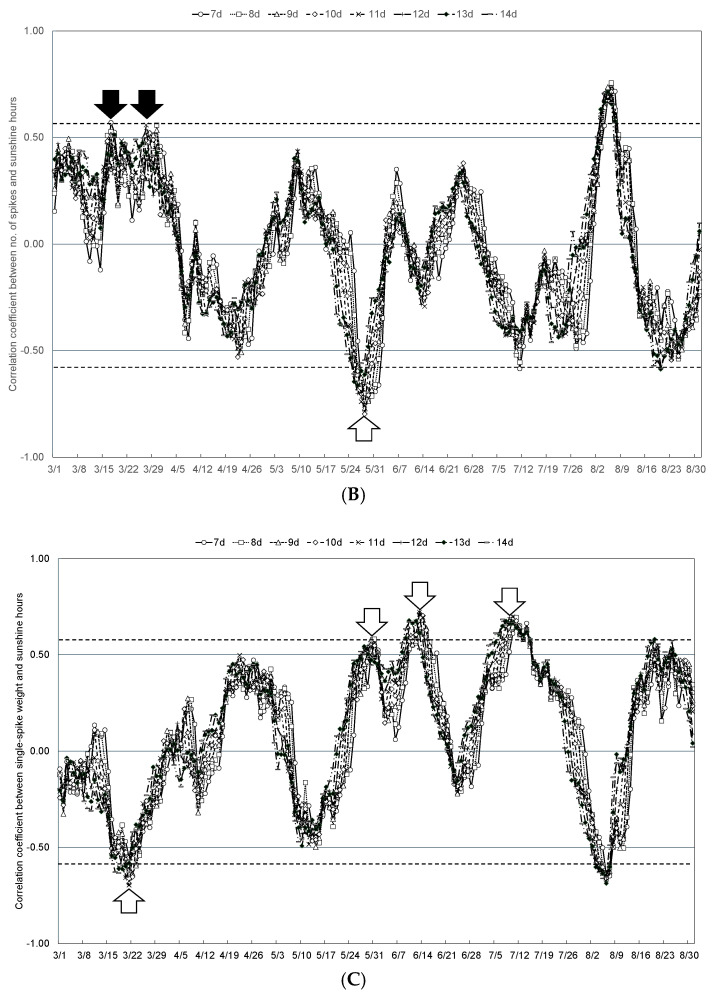

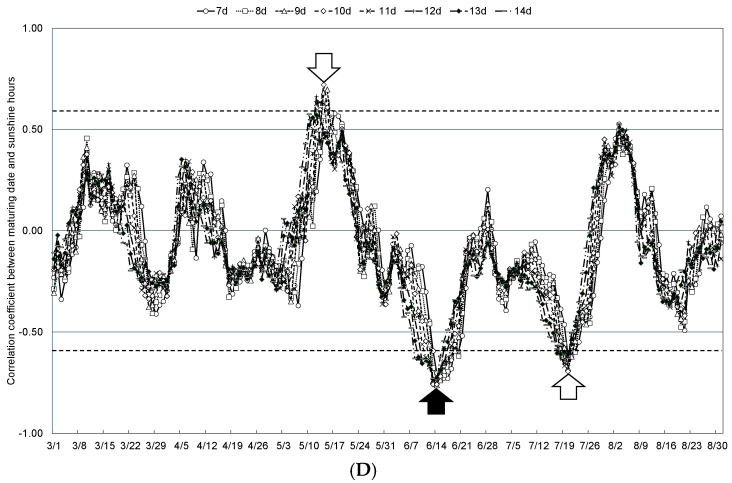
Correlation coefficients between the moving mean sunshine hours over 7 to 14 days and mean wheat yield in the Tokachi Plain (**A**), number of spikes (**B**), single-spike weight (**C**), and maturing date (**D**) from 2011 to 2023. Plots above the dashed line for positive values or below the dashed line for negative values indicate statistical significance at the 1% level. White arrows indicate weather conditions that are statistically significantly correlated, and black arrows indicate weather conditions that were selected as statistically significant explanatory variables.

The relationship between mean wheat yield and weather conditions in the Tokachi Plain from 2011 to 2023, when Kitahonami accounted for more than 90% of the wheat varieties cultivated in the region, was examined by comparing the mean temperatures during any 7- to 14-day periods from spring (March, when the snow-cover period ends) to summer (August, when wheat reaches maturity). The moving coefficients of correlation with mean temperature, mean precipitation, and mean sunshine hours are shown in Figure 5, Figure 6 and Figure 7, respectively. In addition, Table 2 shows the start date and duration of specific periods when mean wheat yield in the Tokachi Plain was significantly correlated with mean temperature, mean precipitation, and mean sunshine hours. Specifically, mean wheat yield showed significant positive correlations with mean temperature for 13 days starting from 27 April during the jointing stage and for 10 days from 19 July during the maturation period. This parameter was also significantly positively correlated with precipitation for 8 days from 27 March and 15 April, at the end of the snow-cover period, for 9 days from 11 June during the flowering period, and for 11 days from 20 July during the maturity period. Significant negative correlations were observed with precipitation for the 9 days from 11 June during the flowering period and for the 11 days from 20 July during the maturing period. Mean wheat yield also showed a significantly negative and positive correlation with sunshine hours for 8 days from 21 March (end of the snow-cover period) and 12 days from 13 June (start of the flowering period), respectively.

Multiple regression analysis was conducted based on the method of decreasing variables (significance level *p* = 0.01) using mean wheat yield in the Tokachi Plain as the objective variable and the weather conditions that were significantly correlated with the above wheat yields (Table 2) as explanatory variables. As a result, three weather conditions were selected: precipitation for 8 days from 27 March, sunshine hours for 8 days from 21 March, and sunshine hours for 12 days from 13 June, yielding the following regression equation:

Wheat yield (kg/10a) in the Tokachi Plain = 37.96 × (precipitation during 8 days from 27 March) − 26.71 × (sunshine hours during 8 days from 21 March) + 36.00 × (sunshine during 12 days from 13 June) + 578.30

The coefficient of determination, adjusted for degrees of freedom, in this regression equation was 0.953, indicating that approximately 95% of the variation in wheat yield in the Tokachi Plain was explained by precipitation during the snowmelt period in late March, sunshine hours in late March, and sunshine hours during the flowering period in mid-June.

Multiple regression analysis was conducted based on the reduced variable method (*p* = 0.01 level of significance) using spike number (i.e., the yield component selected as the explanatory variable for mean wheat yield) as the objective variable and the weather conditions that showed a significant correlation with spike number (Table 2) as the explanatory variables. As a result, three weather conditions were selected, i.e., sunshine hours for 11 days from 27 March, mean temperature for 12 days from 23 April, and precipitation for 11 days from June 14, yielding the following regression equation:

Number of spikes (number of spikes per m2) = 45.56 × (sunshine hours during 11 days from 27 March) − 31.05 × (mean temperature during 12 days from 23 April) + 17.94 × (precipitation during 11 days from 14 June) + 652.56

The coefficient of determination adjusted for degrees of freedom in this regression equation was 0.844, indicating that approximately 84% of the variation in ear number was explained by sunshine hours after snowmelt in late March, mean temperature during the jointing period in late April, and sunshine hours during the flowering period in mid-June.

In addition, multiple regression analysis based on the method of decreasing variables was conducted using single spike weight (i.e., the yield component selected as explanatory variable for mean wheat yield in the Tokachi Plain) as the objective variable and the weather conditions that were significantly correlated with single spike weight (Table 2) as explanatory variables. Considering that only a single explanatory variable was selected at the 1% significance level, this analysis was conducted at the 5% significance level. As a result, the following regression equation was obtained using mean temperature for 13 days from 27 April, precipitation for 9 days from 11 June, and precipitation for 14 days from 7 July:

Single spike weight (g) = 0.0574 × (mean temperature for 13 days from 27 April) − 0.0399 × (precipitation for 9 days from 11 June) − 0.0443 × (precipitation for 14 days from 7 July) + 0.7006

The coefficient of determination, adjusted for degrees of freedom, in this regression equation was 0.786, indicating that approximately 79% of the variation in single-spike weight was explained by the mean temperature during the jointing period in late April, the flowering period in mid-June, and precipitation during the ripening period in mid-July, among other climatic conditions.

The analysis revealed that the weather conditions most significantly influencing wheat yield and its key components in the Tokachi Plain during the period examined were commonly sunshine hours and precipitation in late March (right after the end of the snow-cover period) and in mid-June (during the flowering period). These periods correspond to when overwintered wheat begins rapid vegetative growth to increase tiller number and when immature seeds, which will become the harvested component, start to develop and quickly enlarge after flowering and pollination. Both the vegetative and reproductive growth phases are highly sensitive to precipitation at their onset.

## 3. Discussion

The mean yields of winter wheat in Hokkaido have continued to increase over the long term. However, with the recent climate warming, especially since the 2010s, yields have significantly fluctuated. The relationship between wheat yield and weather in Hokkaido, and specifically in the Tokachi Plain, has been continuously studied [10,11,12,13,14,15]. Record high summer temperatures in Tokachi, such as those observed in 2010, cause serious damage to crop yields. Nishio et al. [13] reported that the record high summer temperatures registered during the summer of 2010 caused major wheat losses in Hokkaido. The study analyzed the relationship between weather conditions and yield components in the cultivars Hokushin and Kitanokaori (the major wheat varieties grown at the time) to clarify the factors contributing to the large reduction in yield. It was revealed that thousand-grain weight, an important wheat yield component, showed a strong negative correlation with mean temperature during the grain-filling period (as similarly reported for foreign production areas) and that the duration of this period also exhibited a strong negative correlation with mean temperature. Shimoda et al. [11] demonstrated that the adverse impact of high temperatures on the grain-filling period in wheat in Hokkaido was alleviated by extended sunshine hours. Specifically, the study elucidated why the negative effect of high temperatures was less pronounced in the Okhotsk region of northeastern Hokkaido, which is more exposed to sunshine, compared with the Tokachi Plain, which receives less sunlight.

Shiga [16] modified WOFOST, a crop model for predicting PY developed in Europe, for application in Hokkaido where the snow-cover period is longer, and devised a highly accurate method to predict the maximum PY of wheat in this region. About 83% of the total variation in PY (without considering water deficit) was explained by the duration of the grain-filling period and solar radiation in July and by the maximum leaf area index. Murakami et al. [17] also developed a machine-learning-based method for predicting wheat yield in each municipality of Hokkaido, which allowed the modeling of wheat yield with a relatively high accuracy using a partial least squares model. Contrariwise, while these crop models and machine learning analyses can tell us when and to what extent each weather condition affects wheat yield and its components, they cannot clearly explain the reasons and mechanisms underlying these effects.

Yanagisawa [15] analyzed the relationship between weather conditions by season (every 10 days) and mean wheat yield in the Tokachi Plain for three representative wheat varieties in Hokkaido from the late 1970s to 2010. The results showed that the total wheat yield over the past 40 years was positively correlated with spring temperatures from April to May, but such a clear relationship was not observed for the older varieties, which were two or three generations before the cultivar Kitahonami used in the present study. In contrast, the yield of the cultivar Hokushin, one generation before Kitahonami (grown from 1997 to 2010), showed a positive and strongly negative correlation with temperature in spring (April) and summer (July and August), respectively, and a strong positive correlation with solar radiation in June and July during the grain-filling period. A trend toward greater yield variability (partial regression coefficient) with changes in temperature and solar radiation was observed for newer varieties, although the mean yield increased. Furthermore, after the 2010s, when the effects of global warming became more apparent in Hokkaido, wheat yields decreased significantly due to high temperatures in June and July during the grain-filling period [13].

Lobbell et al. [8] reported that the most significant effect of global warming on wheat is the shortening of the grain-filling-period toward the end of the growing season, when high temperatures are common in many parts of the world. Nishio et al. [13] conducted a 15-year field experiment in the Tokachi Plain and reported a 6.0% decrease in grain weight and shortening of the grain-filling period by 3.1 days in the wheat variety Hokushin for each 1.0 °C increase in temperature. All previous studies of wheat yield in Hokkaido and Tokachi have been conducted using weather data from April onward. However, with the recent warming, the end the snow-cover period in Hokkaido has moved forward to March, which is an issue that needs to be addressed. For the first time, the present study showed that weather conditions in late March in Tokachi have a significant impact on wheat yield. Therefore, it should be noted once again that the earlier than usual end of the snow-cover period due to global warming strongly affects wheat yield and its components.

Conversely, unlike previous reports, this study reported the maintenance of high yields for the cultivar Kitahonami even when the maturity phase was accelerated due to high summer temperatures. Therefore, a multiple regression (*p* = 0.05) analysis was conducted based on the reduced variable method using the maturity date as the objective variable and the weather conditions that showed a significant correlation with it as explanatory variables. Specifically, these were precipitation for 13 days from 19 March and sunshine hours for 9 days from 14 June. The following regression equation was therefore obtained:

Days to maturity (days from 1 January) = −2.790 × (precipitation for 13 days from 19 March) − 1.371 × (sunshine hours for 9 days from 14 June) + 212.5876

The coefficient of determination, adjusted for degrees of freedom, in this equation was 0.924, indicating that precipitation in late March and sunshine hours in mid-June explained approximately 92% of the variation in the maturity date. Previous reports have shown that the grain-filling period in winter wheat is strongly negatively correlated with mean temperature [4,13]. A strong negative correlation between the number of days to maturity and mean temperature during the grain-filling period (r = −0.806, *p* < 0.01, *n* = 13) was also detected in the present study. However, here, the maturity date in Kitahonami was significantly related to the amount of precipitation in late March during the snowmelt season and to the amount of sunshine in mid-June during the flowering stage. At the same time, these two conditions, which were explanatory variables for the maturity date, were also significant explanatory variables for wheat yield (the positive and negative signs of the explanatory variables are opposite).

If mean temperatures in Hokkaido rise further due to possible future warming, earlier maturation, as observed in 2023, will occur more frequently. In fact, the mean temperature during the wheat grain-filling period in 2023 was 18.76 °C, which is 1.16 °C higher than the mean value over the previous 13 years, and the grain-filling period lasted only 43 days, which is about 6 days less than the mean of 49.2 days in the previous 13 years. However, 6.20 h of sunshine were recorded daily during the 2023 grain-filling period, compared with the mean 4.56 h over the previous 13 years. Therefore, the maturity date in Kitahonami wheat grown at the TAES was 12 July, the earliest in recorded history, and even though the grain-filling period was much shorter than the mean, the yield was 658.6 kg/10a, exceeding the mean yield in the previous 13 years (542.5 kg) by more than 100 kg.

By examining the 20-year period since 2004, this study showed that the 5-year moving mean of the heading period of Kitahonami has shifted to about 8 days earlier, from 9 June to 1 June, and the same maturity date moved to about 5 days earlier, from 24 July to 19 July. Because more days were spent earlier in the heading period than in the maturity date, the duration of the grain-filling period increased by about 4 days, from 44.4 days to about 48.2 days. By comparing the mean temperatures during the grain-filling period from heading to maturity, it was shown that it increased by about 0.7 °C, from 17.06 °C to 17.75 °C. These results indicated that warming is advancing the growth of overwintered wheat and the onset of the grain-filling period, but temperatures during this period are still increasing. In contrast, if the wheat growth phase begins earlier in the Tokachi Plain, plants may avoid the declining sunlight starting from late June onward, and yields may be ensured despite record high temperatures, as in 2023. While the results of this analysis offer some hope for dealing with the possible future warming, further investigations must be conducted, as the timing of high temperatures and low sunshine may be accelerated with warming and seasonal advance.

In this study, for the first time, a detailed day-by-day analysis of the effects of temperature, precipitation, and sunshine duration on wheat growth and yield, as well as its components, was conducted to explore potential adaptive measures against global warming applicable to wheat cultivation in Hokkaido, Japan. The analysis revealed that an excessive number of spikes in the cultivar Kitahonami, which contributes to high yield by increasing the number of tillers, conversely results in low yield when the number of spikes is excessive. In addition, high precipitation and low sunshine conditions in late March immediately after snowmelt tended to result in higher yields. This is thought to be due to these weather conditions in early spring preventing the growth of an excessive number of tillers, which would result in a spike count that is less prone to collapse and in a small grain size. As similarly reported in Shimoda et al. [11] and Nishio et al. [13], this study found that precipitation during the wheat flowering stage had a strong negative effect on yield. In addition, another study of wheat in northern Kyushu, where there is no snow-cover period, showed that precipitation had a strong negative effect on plants at the onset of growth, about 2 weeks after germination, when vegetative growth is active [12]. Similarly, in this study, wheat in Tokachi was shown to be significantly affected by precipitation during the vegetative growth period immediately after overwintering. These results indicated that wheat is generally strongly affected by weather stresses such as precipitation during the rapid growth phase.

Contrariwise, our analysis also showed that the negative effects of high temperatures during the grain-filling period, as reported in Nishio et al. [13] and Yanagisawa [15], were not as strong as before. One possible reason for this is that the advancement of wheat growth has extended the grain-filling period. However, current studies have not fully elucidated whether the future availability of sunshine hours will contribute to counteracting the negative effects of high temperatures caused by global warming.

The results of this analysis show a different climatic response to the relationship between wheat yield and global warming in Hokkaido than has been reported in the past, and the previously known relationship between weather conditions such as temperature, precipitation, and sunlight and yield is partially no longer applicable. In particular, as the crop growth season advances due to warming, the heading period moves forward more significantly than the maturity date, extending the duration of grain filling, and the negative correlation between this parameter and mean temperature, which has a significant impact on yield, tends to weaken. Even in the case of 2023, when the grain-filling period was significantly shortened due to extremely high temperatures, yields were not greatly reduced if certain conditions (such as sufficient sunshine hours) were met, and they were actually higher than the past mean. If wheat growth continues to move forward due to global warming, the correspondence between growth stages and weather conditions is likely to shift in the future. Of particular importance for the successful adaptation of Hokkaido wheat to global warming will be how to secure sunshine hours during the advancing growing season. In addition, warming may lead to a level of growth advancement never experienced before, and previously observed crop responses to weather may no longer apply due to unknown gene–environment interactions. Specifically, in wheat cultivars, variations in the types of alleles of low temperature requirement and genes that are sensitive to the photoperiod may result in nonlinear changes in growth seasons (for example, in terms of heading, maturity date, and grain-filling period) and should be carefully monitored in the future. To ensure the adaptation of wheat production to future warming, it is important to continue to accumulate crop growth and weather data, as in this analysis, and to continue to carefully monitor the effects of climatic changes. By accumulating data using crop growth evaluation and yield mapping from production sites using smart agriculture technology, which has been developing rapidly in recent years, it is expected that the signs of global warming will be detected and dealt with as early as possible and that improved measures to combat this ongoing challenge will be achieved.

## 4. Materials and Methods

### 4.1. Wheat Yield and Yield Component Data

Wheat yield and weather data were collected from the Tokachi Plain, the primary wheat production area in Hokkaido, Japan (Figure 2). Situated on the Pacific Ocean side of Hokkaido, the Tokachi Plain experiences high temperatures, humidity, and reduced sunlight in summer and is influenced by the Chishima Current (a cold current from the northeast) and the Pacific Ocean monsoon during the wheat grain-filling period. As of 2023, this region comprises 19 municipalities, with a wheat cultivation area of 44,572 hectares and a harvest of 293,560 tons [3,18]. Wheat production in Tokachi accounts for approximately 33.7% and 19.4% of the total cultivated area and for 40.9% and 27.0% of the total harvest in Hokkaido and the whole of Japan, respectively. Data on yield components, such as spike number and thousand-grain weight, were obtained from cropping tests on the cultivar Kitahonami conducted at the Tokachi Agricultural Experiment Station (TAES) in Memuro, Hokkaido (42.89° N, 143.08° E). The planting conditions at the TAES were as follows: seeding on September 22 ± 2 days, maize being previously cultivated and here used as green manure, a seeding rate of 255 grains per m^2^, a row width of 30 cm, and four replications of 9.6 m^2^ per plot. The dosage of applied starter fertilizer was 4 g of nitrogen, 16 g of P_2_O_5_, 9.6 g of K_2_O, and 4 g of MgO per m^2^. Additional nitrogen fertilizer was applied at 8 g per m2 in mid-April and 4 g per square meter in late May. Mean wheat yields in the Tokachi Plain were obtained from the data for each municipality provided by the Ministry of Agriculture, Forestry and Fisheries of Japan [1,10]. Climatic conditions, mean wheat yield, and yield components were analyzed over a period of 13 years, from 2011 to 2023, during which Kitahonami accounted for approximately 90% of the wheat cultivation in Tokachi. In addition, yield components for this cultivar were also analyzed over a more extended period of 20 years, from 2004 to 2023, for which growth data were available.

### 4.2. Meteorological Data and Statistical Analysis

Meteorological data, i.e., daily mean temperature, precipitation, and sunshine hours, were obtained from the automated meteorological data acquisition system (AMeDAS) station in Obihiro, which is a weather observation station of the Japan Meteorological Agency located in the center of the Tokachi Plain (Figure 2). Daily snow depth observations recorded at Memuro station (the nearest AMeDAS station to the TAES) were used to determine the start and end dates of the snow-cover period. Microsoft Excel 2021, Excel Statistics Version 4.04, and EZR 1.6.1 [19] were used for the statistical analysis of meteorological data (mean temperature, precipitation, and sunshine hours), wheat yield components, and mean yield during the growing season. Correlation analysis was conducted between mean wheat yield in the Tokachi Plain, mean wheat yield, heading date, maturing date, culm length, spike length, No. of spikes, thousand kernel weight, and No. of kernels per spike at the TAES. To identify the time of the year when weather conditions showed the strongest correlation with mean wheat yield or yield components, the moving coefficients of correlation with mean temperature, mean precipitation, and mean sunshine hours were calculated for any 7- to 14-day period from March to August. These 6 months corresponded to the spring season (when the snow-cover period ends) and the summer season (when wheat reaches maturity) in Tokachi, and the correlation was maximized. The time of the year and number of days were identified. The explanatory variables in the multiple regression analysis of wheat yield and weather conditions were selected based on the 1% or 5% significance level using a stepwise method. In this study, correlations at these two levels were considered as significant.

## 5. Conclusions

(1) Impact of high temperatures on grain-filling and yield stability: high temperatures shorten the grain-filling period, reducing yield, but extended sunshine hours during this phase can lessen these negative effects.
(2) Advancing growth phases: Warming is causing wheat growth stages to occur earlier, extending the grain-filling period and supporting yield stability despite rising temperatures.
(3) Importance of adaptation strategies: As warming advances the growing season, traditional weather–yield relationships are changing. Ensuring adequate sunlight and using smart agriculture to monitor growth and climate data will enable early detection of warming impacts and support timely adaptation.

## Figures and Tables

**Table 1:** Correlation coefficient between mean wheat yield in the Tokachi Plain (TP) and wheat yield components at the Tokachi agricultural Experimental Station (TAES).

	TP Mean Yield (kg/10a)	TAES Yield (kg/10a)	Heading Date (days)	Maturing Date (days)	Culm Length (cm)	Spike Length (cm)	No. of Spikes Per m^2^	Thousand Kernel Weight (g)	Test Weight (g)	No. of Kernel Per Spike
TAES yield (kg/10a)	0.890 **									
Heading date (days)	−0.354	−0.164								
Maturing date (days)	−0.728 **	−0.802 **	0.203							
Culm length (cm)	−0.432	−0.143	0.414	0.301						
Spike length (cm)	−0.216	−0.118	0.420	0.213	0.130					
No. of spikes per m^2^	−0.425	−0.168	0.093	0.291	0.600 *	0.478				
Thousand kernel weight (g)	0.470	0.146	−0.503	−0.188	−0.747 **	−0.592 *	−0.619 *			
Test weight (g)	0.409	0.115	−0.097	−0.099	−0.640 *	−0.301	−0.530	0.851 **		
No. of kernel per spike	0.794 **	0.787 **	−0.031	−0.711 **	−0.325	−0.236	−0.669 *	0.247	0.230	
Single spike weight (g)	0.839 **	0.734 **	−0.191	−0.680 *	−0.499	−0.408	−0.771 **	0.527	0.462	0.952 **

* *p* < 0.05, ** *p* < 0.01.

**Table 2:** List of periods showing the strongest correlation between average wheat yield and the yield components and temperature, precipitation, and sunshine hours in the Tokachi Plain and TAES.

Traits vs. Temperature					Traits vs. Precipitations				Traits vs. Sunshine hours			
	Start Date	Duration	r	*p* Value	Start Date	Duration	r	*p* Value	Start Date	Duration	r	*p* Value
**Mean wheat yield in the Tokachi Plain (kg/10a)**	27-April	13 days	0.635	<0.05	**27-March**	**8 days**	**0.595**	**<0.05**	**21-March**	**8 days**	**−0.789**	**<0.01**
	19-July	10 days	0.694	<0.05	15-April	8 days	0.724	<0.01	**13-June**	**12 days**	**0.885**	**<0.01**
					11-June	9 days	−0.840	<0.01				
					20-July	11 days	−0.568	<0.05				
	Start date	duration	r	*p* value	Start date	duration	r	*p* value	Start date	duration	r	*p* value
**Number of spikes per m^2^**	**23-April**	**12 days**	**−0.694**	**<0.01**	3-April	14 days	−0.742	<0.01	17-March	7 days	0.569	<0.05
	29-May	9days	−0.765	<0.01	14-May	8 days	−0.684	<0.01	**27-March**	**11 days**	**0.559**	**<0.05**
					**14-June**	**11 days**	**0.624**	**<0.05**	28-May	10 days	−0.797	<0.01
	Start date	duration	r	*p* value	Start date	duration	r	*p* value	Start date	duration	r	*p* value
**Single-spike weight** **(g)**	**27-April**	**13 days**	**0.585**	**<0.05**	15-April	8 days	0.732	<0.01	21-March	11 days	−0.696	0.01
	11-July	14 days	0.722	<0.01	**11-June**	**9 days**	**−0.777**	**<0.01**	30-May	9 days	0.580	<0.05
					**7-July**	**14 days**	**−0.622**	**<0.05**	13-June	12 days	0.718	<0.01
									9-July	12 days	0.693	<0.01
	Start date	duration	r	*p* value	Start date	duration	r	*p* value	Start date	duration	r	*p* value
**Maturing date** **(days)**	11-March	14 days	−0.667	<0.05	**19-March**	**13 days**	**−0.683**	**<0.05**	14-May	10 days	0.721	0.01
	1-April	14 days	−0.560	<0.05	2-May	8 days	0.660	<0.05	**14-June**	**9 days**	**−0.762**	**0.01**
	13-June	14 days	−0.841	<0.01	8-June	14 days	0.841	<0.01	20-July	10 days	−0.694	0.01
	22-July	7 days	−0.780	<0.01	20-June	7 days	0.780	<0.01				

The weather conditions adopted as explanatory variables for each of the objective variables (mean wheat yield in the Tokachi Plain, number of spikes, and single-spike weight in TAES) are shown in bold.

## Data Availability

Wheat yield data and weather data can be obtained from the following links. Ministry of Agriculture, Forestry and Fisheries: https://www.maff.go.jp/j/tokei/kouhyou/sakumotu/ (accessed on 7 November 2024). Japan Meteorological Agency: https://www.data.jma.go.jp/stats/etrn/ (accessed on 7 November 2024).
